# Screening for age-related hearing loss in general practice: feasibility, diagnostic Value, and subsequent care pathways–a pilot study

**DOI:** 10.3389/fmed.2026.1872805

**Published:** 2026-07-22

**Authors:** Martin J. Koch, Rebecca Betz, Peter K. Kurotschka, Anne Simmenroth

**Affiliations:** Department of General Practice, University Hospital Würzburg, Würzburg, Germany

**Keywords:** care pathways, diagnostic value, feasibility, general practice, hearing loss, screening

## Abstract

**Introduction:**

Hearing loss (hypoacusis) is highly prevalent among older adults, affecting approximately 33% of individuals aged 65 years and above. It primarily impairs communication and is associated with mental health conditions, dementia, and social impairment. Although various screening methods exist, they are not routinely implemented in general practice. This study aimed to assess the feasibility and diagnostic value of screening for age-related hearing loss in general practices, as well as subsequent care pathways.

**Methods:**

In six general practices, 108 participants aged 65 years and older were screened using easyScreen, a method based on auditory brainstem responses, and were followed up after three and 6 months regarding further diagnostic evaluation and hearing aid uptake.

**Results:**

The screening procedure was brief, well tolerated, and largely compatible with practice workflows. easyScreen detected additional cases compared to questionnaire-based screening alone. The acceptance rates for further diagnostic evaluation and for the recommendation of a hearing aid were each approximately two-thirds.

**Discussion:**

While screening appears feasible and diagnostically valuable, the main challenges lie in post-screening care, particularly in diagnostic follow-up and treatment uptake.

## 1 Introduction

Hearing loss (hypoacusis) is a reduction in hearing ability defined by an increase in the hearing threshold to at least 20 dB HL in the main speech range between 0.5 and 4 kHz (1). The WHO estimates an overall prevalence of hearing loss of 20%, with a higher prevalence of 33% among those aged 65 and older (2). Estimates for Germany range from 16.2% to 40.6%, with older age groups showing a higher prevalence (3–5). Assumed causes include unsafe listening practices (6), ototoxic or viral exposure, chronic illness (e.g., hypertension, diabetes mellitus, central obesity, otosclerosis), smoking, or sensorineural degeneration (2).

The primary symptom of age-related hearing loss is impaired communication due to a loss of speech comprehension, particularly in the presence of background noise. In older adults, hearing loss is a risk factor for mental health conditions such as depression or anxiety disorders and, according to the World Health Organization, may account for up to 8% of dementia cases (2). It also increases the risk of physical health conditions (risk of falls) and social consequences such as a low quality of life, social isolation, and loneliness (2, 7). In an observational prospective study on adults 50 years and older (8), the average time with hearing loss until treatment was more than 10 years and correlated negatively with cognitive abilities. The consequences of age-related hearing loss can be mitigated through early diagnosis and treatment with hearing aids.

To date, there is no systematic screening for hearing loss in the elderly population in most countries. In 2012, only 32 of 76 questioned countries worldwide reported they had implemented regulations or plans for ear and hearing care (9). While no research comparing guidelines, regulations, and plans for screening for hearing loss in older adults exists, a systematic review from 2022 compares newborn and childhood hearing screening (10).

In Germany, there is no systematic screening either in primary care practices or at other points of contact with the healthcare system. Only as part of a basic geriatric assessment, individuals aged 70 and over with a geriatric-specific comorbidity are asked whether they experience difficulty understanding conversations (11). At the same time, the WHO points out that screening for hearing loss and the provision of hearing aids are associated with a significant improvement in hearing-related health outcomes (2). Further, screening and interventions from the age of 65 seem to be cost-effective and harmless compared to the follow-up cost of untreated hearing loss (2, 12).

Electrophysiological devices used for hearing loss screening detect auditory brainstem responses and/or otoacoustic emissions (e.g., the easyScreen used in this study uses auditory brainstem responses only). Auditory brainstem responses are the electrical responses of the auditory brainstem resulting from the conversion of mechanical vibrations into electrical neural signals. Otoacoustic emissions measure sounds in the ear canal that are generated by outer hair cells. They can be used across the lifespan, from newborns to older adults. The BERAphone, the predecessor of the easyScreen device used in this study, is seen as a reliable device in newborn hearing screening. In newborns, the BERAphone showed a specificity of 97.9% and a sensitivity of 100% (13). Other screening methods include pure-tone screening devices, single questions, whisper tests, mobile applications, and questionnaires; the Hearing Handicap Inventory for the Elderly - Screening Version (HHIE-S) was most commonly used. A meta-analysis of thirty-four diagnostic test accuracy studies including adults aged 50 years or older (*n* = 23,228 participants) (14) found that the HHIE-S had a sensitivity for detecting mild hearing loss (> 25 dB HL) between 34% and 58% and a specificity from 76% to 95%. For detecting moderate hearing loss (> 40 dB HL), the pooled sensitivity was 68%, and the pooled specificity was 78%. A HHIE-S cut-off score > 8 was used. In contrast, screening with pure-tone-based screening devices showed a sensitivity ranging from 64% to 93% and a specificity from 70% to 91% for mild hearing loss. For moderate hearing loss, the sensitivity of those devices was high (94%–100%), but their specificity strongly varied (24%–80%). Electrophysiological screening methods were not included in this meta-analysis.

Although the diagnostic value of hearing-loss screening is well established, the feasibility of implementing large-scale programs in older adults remains uncertain. In contrast, newborn hearing screening is routine in many high-income countries; for example, Germany has required universal electrophysiological screening for all infants since 2009 (15), and the programme fulfils the classic criteria for a “good” screening test (16) – it detects a treatable condition early, is inexpensive, painless, and can be completed within minutes. EasyScreen extends these advantages to older adults. It targets an important health problem with a prevalence of 33% for those above 65 years of age that is a risk factor for depression, falls, dementia, and loneliness. It can be detected at an early, largely asymptomatic stage. It is safe and easy to administer with little training, may offer high sensitivity and reasonable specificity, is adequately costed, and can lead to effective treatment in older adults. An international systematic review of screening tools for older adults concluded that several instruments (including pure-tone audiometry, self-report questionnaires, and handheld devices) have adequate sensitivity and specificity for detecting age-related hearing loss (14). The authors noted the possibility of harm (e.g., false positive results that lead to unnecessary testing/treatment, labeling, or anxiety) but report that no studies have been conducted on these.

General practices provide accessible and frequent contact with older adults, especially those with multimorbidity, allowing screening to be integrated into routine care with support from primary care staff and limited disruption to existing workflows (17–19). Long-standing patient–physician relationships might motivate patients (and their relatives) to seek further diagnostic evaluation and treatment. In Germany, 90% of the population has public health insurance, which covers consultations and diagnostics in primary care and otorhinolaryngology. Moreover, most hearing care professionals provide initial hearing assessments at no cost. Reimbursement for hearing aids, however, requires a referral to an ENT specialist for a formal prescription. Patients are entitled to a new hearing aid with approximately €750 (per single device) every 6 years and a copayment of €20. If a higher-end device is desired, the difference must be paid out of pocket.

Older patients often consider hearing loss as a normal part of ageing that is untreatable with low daily impairment. Aid is only sought as a “last resort,” and devices are often perceived as ineffective and inconvenient (20). Relatedly, many elderly adults stigmatize hearing loss and fear stigmatization when wearing hearing aids (21, 22). A substantial proportion of individuals who screen positive for hearing loss and are advised to undergo further diagnostic evaluation or treatment do not follow through with these recommendations. Even among those who seek treatment, uptake of hearing aids remains limited: a 2023 meta-analysis found that approximately 38 % of individuals who owned a hearing aid did not use it (23). Among hearing aid users, device-related problems were frequently reported (48.3%), while among non-users, a common reason for not using the device was that hearing aids had not been recommended to them (33.5%) (22).

A recently published study protocol (24) outlines an approach to evaluate the feasibility of a hearing program that includes counseling on alternative hearing rehabilitation strategies. However, the focus lies on a specific intervention and not on the screening and subsequent care pathways. To the best of our knowledge, to date, no studies have examined the feasibility, diagnostic value, and future direction of hearing loss screening in general practice. To address these gaps, an empirical study was conducted in a general practice setting to answer the following research questions:

1. How feasible is the use of easyScreen in general practice with regard to test duration, testing conditions, technical malfunctions, and the subjective evaluations of general practitioners and medical assistants?
2. What additional diagnostic value does easyScreen provide compared with the HHIE-S in terms of identifying hearing loss cases and prompting further diagnostic evaluations?
3. What care pathways do participants follow after screening, up to and including hearing aid use?

## 2 Materials and methods

### 2.1 Study design and participants

We conducted a prospective, observational, exploratory pilot study in six general practices in Würzburg and the surrounding county. In October 2023, practices affiliated with the Bavarian Research Practice Network (BayFoNet) and academic teaching practices cooperating with our department were informed about the study verbally during a training course and subsequently invited via letter. Practices that expressed interest in participating received further information from a member of the study team. Study dates were then arranged in agreement with the respective practices. We assumed that six practices could reasonably be enrolled in this pilot study. Further, we assumed a prevalence of presbycusis of at least 16%, a patient volume of approximately 20 patients who are age 65 or older per practice per day, and an eligibility and willingness to participate of 50% of these patients. We expected that approximately 120 patients could potentially be enrolled during 12 planned screening days (two per practice).

Data collection took place over a 3-month period, from 21 October 2024 to 16 January 2025. Of the participating practices, one was a single-physician practice, while the remaining five had between two and five general practitioners.

Patients aged 65 or older attending the participating practices on the study day were recruited by the study physician. In some practices, patients were invited by the practice team beforehand. Patients were excluded if they met one of the following criteria: current use of a hearing aid or cochlear implant; severe cognitive impairment; a prior diagnosis of hearing loss based on the gold standard (pure-tone audiometry) or current treatment for hearing loss; cerumen obstructing the ear canal; any other ear condition that could impair hearing or interfere with the test performance, such as cholesteatoma.

### 2.2 Study procedure

At baseline, eligibility was assessed. Participants received oral and written study information and provided written informed consent. After consent was obtained, participants completed a baseline questionnaire as well as the HHIE-S. Subsequently, the easyScreen procedure was administered by a member of the study team (95.4%, RB, a medical student acting as the study coordinator). In five cases, a trained medical assistant who was supervised by RB (4.6%) administered the screening. Involving medical assistants in parts of the testing process served to evaluate the feasibility of test administration from the perspective of practice personnel and to identify the conditions required for its routine use. Familiarization with the device required ten minutes or less.

Participants with a positive screening result were informed whether the HHIE-S and/or the easyScreen indicated abnormal findings, and were advised to seek further diagnostic evaluation, preferably pure-tone audiometry, the gold standard. At the same time, we emphasized that this was a pilot study and that, at the time of testing, no reliable estimate could be provided regarding the likelihood of an actual hearing impairment. For patients seeking further evaluation, we advised bringing the study information materials to their audiology appointment. In a small number of cases in which patients expressed concern about recalling the study information accurately, we documented the screening results directly on the provided information sheet. This approach was chosen instead of gold standard testing to reflect the real-world care pathways following a positive screening result.

**FIGURE 1:**
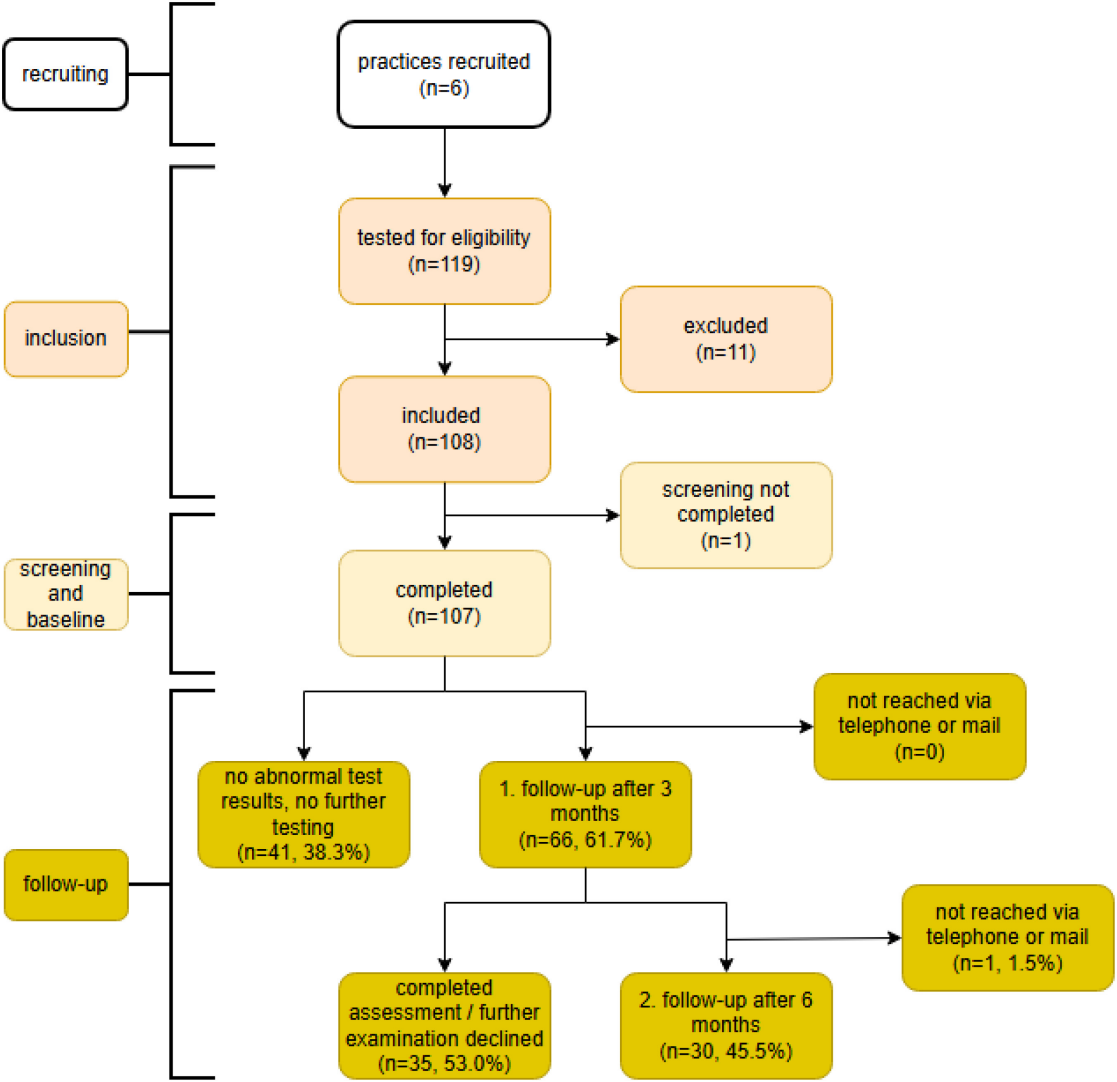
Flow diagram illustrating participant progression.

Participants were followed up via telephone 3 months after enrolment. All phone calls were made by RB. During this follow-up, information was collected on any subsequent diagnostic and therapeutic care, including, where applicable, hearing aid use. If no further care had been initiated by the 3-month follow-up, participants were contacted 3 months thereafter (i.e., at 6 months post screening) and asked the same set of questions. Figure 1 presents the participant flow through the study.

### 2.3 Screening

To screen for hearing loss with easyScreen (MAICO, Germany), electrodes were attached at three positions on the participant’s head, and foam ear inserts containing a probe were placed in both ear canals. The device performs binaural measurements of early auditory brainstem responses. The stimulus consists of click sounds presented at a repetition rate of 90 clicks per second, with frequency components ranging from 2 to 5 kHz. The acoustic stimulus is a chirp signal delivered via sound tube earphones, applied simultaneously to the right and left ears. Further details can be found in the data sheet (25). Test duration varies from 10 to 180 s. A screening result was considered positive if no response or only an insufficient response was detected at 60 dBHL. This stimulus level was defined on the basis of a preliminary study involving 95 adults aged 60 years or older (26). This hearing level was programmed by the manufacturer specifically for our purposes.

The HHIE-S (27, 28) was used as an additional screening instrument to assess self-perceived hearing-related quality of life. The HHIE-S requires approximately three to 5 min to complete. Participants must be cognitively able to judge whether situations occur in which they experience hearing difficulties. The questionnaire comprises 10 items addressing emotional well-being and social or situational limitations. Each item is scored as 0, 2, or 4 points (“no” = 0, “sometimes” = 2, “yes” = 4). In the present study, a total score greater than 8 was considered clinically significant. In a cross-sectional substudy of the Framingham Heart Study, the HHIE-S showed 35% sensitivity and 94% specificity (29).

### 2.4 Survey of practice staff

To evaluate the feasibility of easyScreen, general practitioners and medical assistants from each practice were surveyed using a pen-and-paper questionnaire. The questionnaire included questions on the age of the practice staff, structural data of the practice, and seven questions about their evaluation of the feasibility of easyScreen. They were verbally informed about the purpose of the study and implied consent by completing the questionnaire. The questionnaire was administered at the end of the study procedures in the respective practice. We ensured that each medical assistants had independently administered one hearing test before completing the questionnaire. This was not possible in one practice, presumably due to a high workload. Consequently, in this practice, two general practitioners answered the questionnaire.

### 2.5 Statistical analysis

The statistical analyses were conducted using R version 4.5.2 (30) with RStudio version 2026.1.0.392 (31). Descriptive statistics were calculated to characterize the sample and assess the feasibility of using easyScreen. Means and standard deviations are reported for metric variables. For categorical variables, absolute and relative frequencies are provided.

To assess the diagnostic quality of the screening instruments, the positive predictive value (PPV) was calculated for both easyScreen and HHIE-S. Cases in which both screening methods yielded an abnormal result were counted as positive cases for both methods. Unclear cases were counted as not confirmed for the purpose of this analysis. To estimate the incremental predictive validity of easyScreen, we additionally examined how many true-positive cases would not have been identified without the use of this tool. Furthermore, we analyzed how many cases involved further diagnostic procedures that were initiated due to a false-positive easyScreen result and later proved unnecessary.

A Sankey analysis was performed to describe the subsequent care pathway. This is presented as a Sankey diagram created using the package ggsankey (32). Descriptive statistics (absolute and relative frequencies) are provided for the reasons why recommendations for further diagnostic evaluations and hearing aids are not followed.

## 3 Results

### 3.1 Sample

A total of 108 individuals participated in the study, corresponding to an average of 18 participants per primary care practice (Table 1). Data are reported on all individuals who completed the screening (*n* = 107). The mean age of participants was 72.6 years (*SD* = 5.9), of whom 57.0% identified as females. Most participants were no longer employed (92.5%). Approximately one quarter reported occupational noise exposure without regular use of hearing protection (25.2%).

**TABLE 1:** Sample characteristics.

Variable	Total (*n* = 107)	Hearing loss not detected (*n* = 22)	Hearing loss detected (*n* = 20)	Did not seek diagnostics (*n* = 65)
	M (SD)	M (SD)	M (SD)	M (SD)
Age	72.6 (5.9)	70.6 (4.9)	72.9 (6.0)	73.3 (6.1)
Number of long-term diagnoses	2.2 (1.7)	2.5 (1.5)	2.3 (1.5)	2.1 (1.7)
Number of long-term medications	2.1 (1.5)	2.4 (1.6)	1.8 (1.6)	2.0 (1.4)
	***n* (%)**	***n* (%)**	***n* (%)**	***n* (%)**
Age ≤ 75	73 (68.2%)	18 (81.8%)	14 (70.0%)	41 (63.1%)
Female gender	61 (57.0%)	12 (54.5%)	10 (50.0%)	39 (60.0%)
Academic degree: none	1 (0.9%)	1 (4.5%)	0 (0.0%)	0 (0.0%)
lower secondary	66 (61.7%)	11 (50.0%)	11 (55.0%)	44 (67.7%)
intermediate secondary	26 (24.3%)	7 (31.8%)	6 (30.0%)	13 (20.0%)
higher secondary	14 (13.1%)	3 (13.6%)	3 (15.0%)	8 (12.3%)
Retired	99 (92.5%)	22 (100.0%)	19 (95.0%)	58 (89.2%)
Noise exposure at work	46 (43.0%)	11 (50.0%)	8 (40.0%)	27 (41.5%)
Hearing protection at work	20 (18.7%)	6 (27.3%)	6 (30.0%)	8 (12.3%)
Cardiovascular diseases	70 (65.4%)	16 (72.7%)	13 (65.0%)	41 (63.1%)
Metabolic disorders	54 (50.5%)	13 (59.1%)	12 (60.0%)	29 (44.6%)
Cancer	25 (23.4%)	7 (31.8%)	4 (20.0%)	14 (21.5%)
Other chronic diseases	31 (29.0%)	4 (18.2%)	6 (30.0%)	21 (32.3%)

### 3.2 Screening feasibility and diagnostic value

The easyScreen device was easy to operate. After a brief introduction of no more than 10 min, medical assistants were able to perform all required procedures. Some difficulties arose due to electrical interference, likely caused by nearby electronic devices such as mobile phones or smartwatches. Once removed, the test was administered without major issues.

The screening with easyScreen took a mean of 02:24 min (SD = 00:48). Among the 107 participants, the screening was performed with one participant in a seated position, while all others were examined in a supine position. Approximately one-third of participants kept their eyes closed during the procedure (34.3%). Interfering noise was present in ten cases (9.3%). In one additional case, the screening could not be successfully completed despite a second attempt because of substantial background noise. In all six practices, a separate consultation room was used. Patients had access to an examination couch, and a workspace was available for the study team.

EasyScreen identified abnormal findings in 58 (54.2%) participants. The mean HHIE-S score was 5.1 (SD = 5.4), and 20.6% of participants scored above the predefined clinical cutoff of 8. When both screening methods were considered together, 38.3% of participants had normal results on both tests. In 48.6%, only one of the two screening methods was abnormal, either the HHIE-S (7.5%) or easyScreen (41.1%). In 13.1%, both screening methods showed abnormal results.

Overall, at least one of the two screening instruments was abnormal in 66 participants (61.7%). Of these, 44 (66.7%) underwent further diagnostic evaluation. Among those 44 individuals, 22 (50.0%) were not found to have hearing loss when tested with pure tone audiometry, 20 (45.5%) received a confirmed diagnosis of hearing loss, and two (4.5%) had inconclusive diagnostic results. The positive predictive value (proportion of true positives among all positives) was 48.7% for easyScreen and 46.1% for the HHIE-S (Table 2). Patients that were screened positive by both instruments were considered for both positive predictive values.

**TABLE 2:** Crosstabulation of abnormal findings in screening and diagnosed hearing loss.

	Hearing loss			
	Not confirmed	Confirmed	Unclear	Total
HHIE-S	4	1	0	5
EasyScreen	15	14	2	31
Both abnormal	3	5	0	8
	22	20	2	44

Of the 20 participants with confirmed hearing loss, 14 would not have been identified without easyScreen (70.0%). However, easyScreen also prompted additional diagnostic evaluations in 15 of the 22 participants who were ultimately classified as false positives, meaning that these follow-up assessments proved unnecessary in retrospect (68.2%).

### 3.3 Results of the survey of practice staff

A total of twelve health care providers (two per practice) were surveyed for the study, with a mean age of 35.9 years (*SD* = 11.5). Seven were general practitioners, and five were medical assistants. Half of them had more than 10 years of experience (50.0%). The others had between 1 and 5 years (25.0%) or between 6 and 10 years (16.7%). Some did not report their experience (8.3%).

The survey (Table 3) showed that all twelve respondents considered screening for hearing loss in older adults to be important. Respondents generally agreed that the time required for screening with easyScreen was appropriate, that patients were receptive to the procedure, that the training provided was sufficient, and that screening did not disrupt routine practice workflows. Views on the ease of integration into everyday practice and on continuation beyond the study period were somewhat more variable, although they remained predominantly positive.

**TABLE 3:** Results of the survey of general practitioners and medical assistants.

Variable	disagree	neutral	agree
I believe that screening for hearing loss using easyScreen is important for all older adults.	0.0	0.0	100.0
The easyScreen screening can be easily integrated into the practice’s routine.	16.7	16.7	66.7
I would continue to offer the easyScreen screening in my practice even after the study is completed.	16.7	33.3	50.0
The time required for screening with easyScreen per patient was reasonable.	9.1	0.0	90.9
Patients’ attitudes toward screening with easyScreen were predominantly positive.	16.7	8.3	75.0
The training on how to use the device was sufficient.	0.0	22.2	77.8
Conducting the screening with easyScreen disrupted the practice workflow.	91.7	0.0	8.3

### 3.4 Care pathways after screening

**FIGURE 2:**
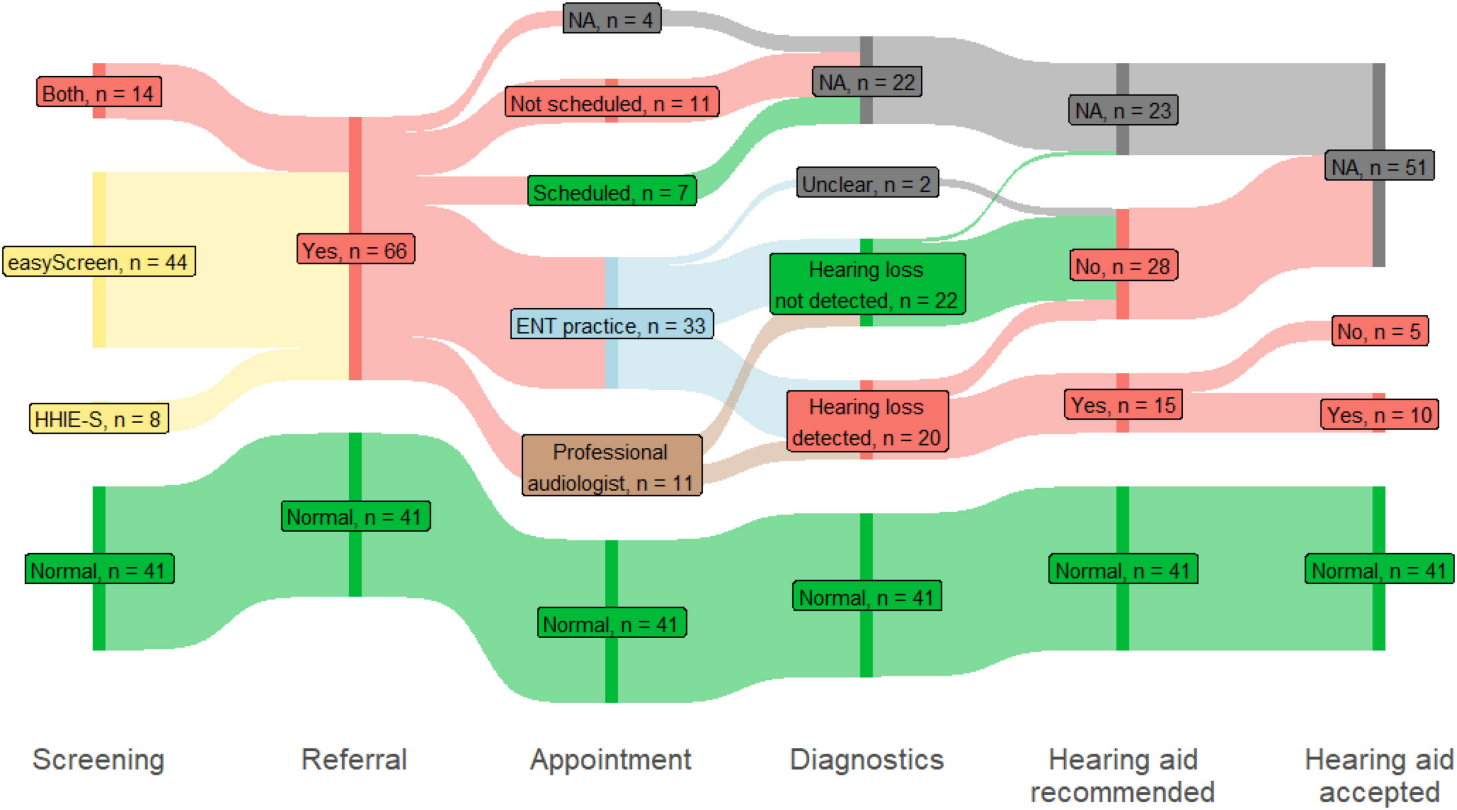
Sankey diagram of care pathways after screening. ENT, otorhinolaryngology. NA, Not available; patients were not further followed, and no data were available on later steps of the care pathways.

Figure 2 shows the care pathways after screening. Of the 107 participants with a completed screening, 66 had an abnormal result in at least one of the two screening procedures and were therefore advised to undergo further diagnostic tests. Of these, 44 attended a diagnostic appointment. For seven additional participants, an appointment had already been scheduled at the time of follow-up, while eleven had not arranged an appointment despite the recommendation. No follow-up information was available for four participants. 13 participants reported why they did not seek further diagnostic evaluation: All except one reported that they did not feel limited in their ability to hear. Three reported they had no time or opportunity for diagnostic evaluation, and one reported that further diagnostic evaluation would not help anyway. Further diagnostic evaluation was conducted mainly by otorhinolaryngologists (*n* = 33) and less often by professional audiologists (*n* = 11). Among the 44 individuals who completed diagnostic evaluation, hearing loss was ruled out in 22, confirmed in 20, and remained unclear in two.

In total, 15 individuals with confirmed hearing loss were advised to use a hearing aid. In five cases, no hearing aid was recommended despite the diagnosis of hearing loss. In both cases with inconclusive diagnostic findings, no recommendation for a hearing aid was made. Of the 15 individuals who received a recommendation for a hearing aid, ten accepted this treatment, while five declined it despite their diagnosed hearing loss. Three participants reported they had no opportunity, and one reported they wanted to seek a second opinion.

## 4 Discussion

The aim of this pilot study was to test the feasibility and provide first evidence of the diagnostic value of a novel screening tool for age-related hearing loss in general practice, and to describe subsequent care pathways. To our knowledge, this is the first study to investigate these questions in a primary care setting.

Our findings suggest that the easyScreen procedure is feasible in general practice. Screening was brief, with an average duration of less than two and a half minutes. Background noise interfered with testing only occasionally, and all but one procedure could be completed. No adverse effects or device-related problems were reported. In addition, general practitioners and medical assistants viewed the screening procedure positively overall. They considered screening for hearing loss in older adults important and generally rated the time required, the training provided, patient acceptance, and the compatibility with routine practice workflows favorably. While prior research shows that screening is cost-effective and can help to improve hearing aid use (2, 12), the current study provides first indications that screening can be integrated into primary care workflows and can be performed by trained medical assistants. This is particularly relevant for German general practitioners, where consultation time is limited and successful implementation often depends on whether tasks can be delegated (33).

EasyScreen showed a higher positive predictive value compared to the HHIE-S. It is particularly noteworthy that a large proportion (70%) of the cases later confirmed as hearing loss by full audiometry (true positives) would not have been detected by the HHIE-S questionnaire alone. This suggests that objective screening may identify individuals who do not report sufficient hearing-related difficulties on self-assessment. In that respect, easyScreen may add diagnostic value beyond perceived hearing impairment. Existing evidence shows considerable variability in the reported accuracy of hearing screening methods, likely because studies differ in their definitions of hearing loss and in the thresholds they apply. Some evidence also suggests that simple approaches, such as a single question (“do you have trouble with hearing or understanding conversations?”) may achieve accuracy comparable to more elaborate screening procedures, but could also be administered too late if it is part of a geriatric assessment (14). In Germany, such a question is currently used only in the presence of age-related comorbidity, mostly in patients 70 or older (11). However, its performance in German general practice would need to be examined directly and compared with objective methods such as easyScreen.

The care pathways observed after screening show that the feasibility of the screening procedure itself does not necessarily translate into successful implementation of the full screening-to-care pathway. Although the test could be administered successfully in practice, a substantial proportion of participants with abnormal screening results did not proceed to further diagnostic evaluation. About one-third of those referred for diagnostic clarification had not arranged an appointment within 6 months. In addition, one-third of those who were advised to use a hearing aid did not accept this recommendation. These findings suggest two major points at which patients are lost along the care pathway, corroborating earlier studies. In our study, participants mainly reported no perceived limitations in their ability to hear and limited time and opportunity as reasons.

Our study suggests that screening alone is unlikely to improve hearing care unless it is embedded in a broader strategy that addresses subsequent diagnostic and therapeutic steps. Future interventions should therefore not only focus exclusively on the screening method itself. They also need to address communication after a positive result, support for arranging diagnostic appointments, and counseling on available rehabilitation options. Alternative rehabilitation strategies (24) or psycho-social interventions (34–36). may be particularly valuable for individuals who are reluctant to use hearing aids or who do not consider their hearing difficulties as treatable. General practices offer accessible, frequent contact combined with long-standing patient-physician relationships (37). This places them in a strong position to initiate screening, support patients in navigating subsequent steps, and encourage them to seek further care.

### 4.1 Strengths and limitations

Our study was conducted in primary care practices and therefore provides data from a clinically relevant setting. It combined information on implementation, screening outcomes, and subsequent care pathways, which allowed us to examine whether screening could be performed and what happened afterward. The procedure can also be performed by trained medical assistants, which offers a realistic indication of how screening might be integrated into everyday care.

The study has important limitations. First, the screening results were not compared with pure-tone audiometry as the gold standard. Instead, further diagnostic evaluation was recommended only to those with abnormal screening test results, and attendance depended on participants’ own decisions. As a result, diagnostic test accuracy metrics could not be estimated, and the positive predictive values could only be calculated in a subsample of participants. Second, the quality and comparability of the diagnostic follow-up could not be controlled because participants chose where to seek further evaluation, for example in an otorhinolaryngology practice or with a professional audiologist. Third, the study was conducted under pilot conditions in a limited number of practices. The results therefore cannot be assumed to represent routine implementation in all general practices. Fourth, the follow-up period of 6 months may have been too short to capture the full care pathway, as six participants had arranged appointments but had not yet attended them by the end of follow-up.

Future research should include larger samples and possibly longer follow-up periods. It would be worth investigating how screening for hearing loss can be embedded in care pathways that reduce attrition after a positive result and improve uptake of recommended treatment. A comparative study should also examine whether simpler approaches, such as a single question on self-perceived hearing loss, may be sufficient in primary care. In addition, future studies should include cost-effectiveness analyses to evaluate the benefits and costs of hearing loss screening.

### 4.2 Conclusion

This pilot study provides first evidence that screening for hearing loss with easyScreen is feasible in German general practice. The procedure was brief, well tolerated, and largely compatible with practice workflows. easyScreen also appeared to identify cases that would have been missed by questionnaire-based screening alone. Given the current state of the evidence, we cannot yet recommend implementing the HHIE-S, easyScreen, or both. However, the added value of the HHIE-S over the easyScreen might be limited. The study also shows that successful implementation cannot be judged by the screening procedure alone. The main challenges may arise after the test, particularly in diagnostic follow-up and treatment uptake. Future studies should therefore evaluate screening for hearing loss in primary care as a complete care pathway rather than as an isolated diagnostic step.

## Data Availability

The raw data supporting the conclusions of this article will be made available by the corresponding author, without undue reservation.
